# Dermatology

**Published:** 1878-06

**Authors:** 


### DERMATOLOGY.

TREATMENT OF BROMIDE OF POTASSIUM ERUPTION. (*Br. Med. Jour.*, March 1878.)—Dr. Russell had a severe case of this trouble in the person of a young woman whom he was treating for epilepsy. The eruption was first papular, then pustular. By combining five minims of Fowler's solution with each dose of the bromide he completely overcame the annoyance and was able to continue the administration of the latter as long as he desired.

TREATMENT OF ACNE ROSACEA. (Archives of Dermatology.)—Narmann recommends a solution of one pt. carbolic acid in three or four parts alcohol, as an application to the diffusely reddened patches. It is not, however, of much service, when there is infiltration or vascular ectasis.

